# Social and Societal Factors Interact with Psychological Factors to Shape Pain Outcomes in a Community Sample with Chronic Pain: A Network Study

**DOI:** 10.1101/2025.08.29.25334446

**Published:** 2025-09-03

**Authors:** Gwen van der Wijk, Lisette de Groot, Joline Bruweleit, Lilla Hévizi, Meredi S. Edgarian, Stijn Vermeulen, Lakeya S. McGill

**Affiliations:** (1) Department of Clinical Psychological Science, University of Maastricht, the Netherlands; (2) Independent Researcher & Community Partner; (3) Department of Medicine, University of Pittsburgh School of Medicine

**Keywords:** chronic pain, network analysis, social-societal factors, psychological factors

## Abstract

Although the biopsychosocial model of chronic pain is widely recognized, few studies include social and societal factors as variables of interest, limiting our understanding of their role and importance in managing chronic pain. In the present study, we examined how social and societal factors interact with psychological factors to shape pain outcomes. We recruited 262 participants with chronic pain from the community (e.g., through the university, online distribution, GP and physiotherapist offices) to collect self-reported data about emotional states (e.g., mood symptoms, positive affect), pain coping strategies (e.g., pain acceptance, pain avoidance), social interactions (e.g., emotional support, pain invalidation), societal stressors (e.g., financial worry, access to health care) and pain outcomes (quality of life, pain disability and pain intensity). In line with previous network studies, our partial correlation network including only psychological factors showed that multiple psychological factors are uniquely associated with pain disability. We then extended the partial correlation network with social and societal factors, and demonstrated that these had strong direct relationships with quality of life, and were indirectly related to pain intensity and pain disability through pain-related worry and pain acceptance. Overall, our results indicate that social and societal factors are important for understanding pain outcomes and should thus be considered in interventions targeting chronic pain, alongside psychological factors. Future work should examine the interactions among social-societal and psychological factors in more depth to inform ways to incorporate this into individual pain management and societal interventions for chronic pain.

## Introduction

Chronic pain is a disabling condition affecting individuals all over the world. In line with global standards, we define chronic pain as persistent or recurrent pain for three months or more [1]. The overall prevalence of chronic pain in Europe is approximately 20%, and studies show an increase in the global prevalence of chronic pain, with even higher numbers expected for certain subtypes in the regions South Asia, Tropical Latin America and North America in the future [2,3]. The prevalence of chronic pain also differs between socio-demographic groups within countries. For example, a systematic review of European studies found a higher prevalence of chronic pain among women, individuals with a low income, poor social support, and Asian and Black groups, stressing the importance of taking sociodemographic dimensions into account [4].

Although sociodemographic variables are associated with chronic pain, they do not inherently explain the underlying causes of differences in its prevalence. The World Health Organization (WHO) addresses this with its notion of ‘social determinants,’ in which they explain that health inequalities arise “*[…] because of the circumstances in which people grow, live, and age, and the systems put in place to deal with illness. The conditions in which people live and die are, in turn, shaped by political, social, and economic forces*” ([5], p. 3). In this article, we use ‘social indicators of health’ to reflect that these circumstances, while influential, do not predetermine any individual’s outcomes but can change and be changed [6]. In line with this perspective, we look at specific social and societal experiences instead of demographic group membership, as this may reveal more direct and useful insights (e.g., looking at experiences of discrimination instead of racial identity; [7].

In the majority of psychological research on chronic pain, such social indicators are not taken into account [8]. Instead, the focus tends to be on individual psychological characteristics. For example, a popular theory of chronic pain is the fear-avoidance model, which states that pain-related worrying, as well as the subsequent pain-related fear and avoidance behaviors, can lead to higher pain disability and worse pain [9]. Many studies have supported this model (e.g., [10,11]), and it has led to the development and implementation of exposure therapy for chronic pain [12]. At a more general level, higher levels of anxious and depressive symptoms have been observed in people experiencing chronic pain, and this has been associated with worse pain outcomes [13,14].

The importance of positive psychological factors in chronic pain is also increasingly recognized [15]. Broadly, the idea is that factors such as positive affect and pain acceptance can help people manage their chronic pain and improve their quality of life by allowing them to stay engaged in activities they value. A prominent theory from this approach is the psychological flexibility model, which focuses on adjusting responses to thoughts and feelings associated with pain instead of trying to change the pain-related thoughts and feelings themselves [16]. For example, it proposes that higher pain acceptance will allow someone to move away from fighting the pain and instead invest their energy into doing valued activities despite the pain. This model has been translated into acceptance and commitment therapy (ACT) for chronic pain [17].

Although many studies show associations between these psychological factors and important pain outcomes, and different forms of psychological treatment targeting these factors have been shown to be effective, treatment effects are generally small to moderate and vary between individuals [18-20]. One potential reason for these limited results may be that the frameworks such treatments are based on largely ignore the social-societal context [8]. Indeed, foundational and recent work from both a sociology and critical medical anthropology perspective indicates that social, cultural and political context is essential for understanding health, as it reflects, is shaped by and deeply embedded in these contexts [21-25]. Bringing this context into the picture may bring a better understanding of the roots of variation in psychological factors, and through that, may reveal ways to make therapy more nuanced and sensitive to the social, cultural and political context individuals navigate [8,26]. At the macro level, examining the relative contributions of individual and contextual factors may provide directions on the most promising targets for reducing chronic pain complaints and inequities.

Partial correlation networks examine the unique relationships between pairs of variables while controlling for all others in the network, and are therefore well suited to explore the complex interactions and relative contributions of a large number of factors. In the context of chronic pain research, most studies have used network analyses to investigate the relationships between psychological and pain variables (e.g., [27-29]). A handful of studies has included one or more social and/or demographic factors next to psychological ones, and found that they are often related to pain intensity and pain disability (e.g., peer problems in adolescents, [30]; perceived injustice in women experiencing vulvodynia, [31]; gender in chronic low back pain, [32,33]. Notably, Gevers-Montero et al. [34] observed that the factors with the strongest connections in their chronic pain network were demographic (age, gender and marital status). While these studies thus suggest that social and societal factors have unique interactions with pain outcomes next to psychological variables, the available evidence is limited.

In the current study, we use network analysis to explore the complex interplay between psychological, social and societal factors and identify how they relate to pain intensity, pain disability and quality of life among people with chronic pain. We collected self-reported data on a wide range of factors from a large community sample of people experiencing chronic pain (*N* = 262) through an online survey. We used cross-sectional regularized networks consisting of partial correlations to estimate unique relationships between all included variables. We first examined if a network containing only fear-avoidance and mood factors in relation to pain intensity and pain disability could replicate previously published network studies [28,29], and then explored what a network with factors from all measured dimensions (psychological, social and societal) could reveal. For the replication network, we expected a dense network of positive relationships, with pain intensity and depressive symptoms being most strongly related to pain disability. For the extended network, we expected that social and societal variables would have unique connections with pain intensity, pain disability and quality of life beyond what psychological variables explain.

## Methods

This study was preregistered on AsPredicted (see https://aspredicted.org/8wys-csn4.pdf) prior to data collection, and subsequently updated prior to data analysis to ensure the reliability of our analyses with our achieved sample size (see https://osf.io/p3djf/?view_only=8f243e0b6eb5481087e4fb12c56f9e70).

### Participants

Participants were recruited at Maastricht University, chronic pain related associations, general practitioners and physiotherapy offices in the Netherlands and Germany, and the authors’ social networks. The survey was first assembled in English, and later translated to both Dutch and German. Participants were eligible if they were 18 years or older, spoke sufficient English, Dutch or German and had experienced persistent or recurrent pain for at least 3 months. All participants received the same survey. There were two incentives for participation. Students from Maastricht University could choose whether to earn 0.5 university credits or to enter a lottery with a 1/50 or higher chance to win a €50 voucher. Non-UM students could choose to enter the lottery. Data collection started in May 2024 and ended in August 2025.

To ensure data quality, the data was cleaned before the analysis. A total of 426 participants provided written informed consent for the study. Individuals who did not meet the inclusion criteria were excluded (*N* = 8 did not experience pain in the past six months, *N* = 12 reported a pain duration of less than three months). We identified and removed 5 duplicates. Next, we removed participants who did not fill out any of the questions beyond the demographics or pain characteristics (*N* = 119). Additionally, we identified and removed participants who finished the survey in less than 5 minutes (*N* = 2). The five minute threshold was chosen since careful completion by the researchers took at least seven minutes in pretesting. In line with recommendations, we planned to remove participants who answered two or more attention checks wrong [35], however, this was not the case for any of our participants. Lastly, participants were excluded if they missed more than 50% of the items for one or more of the variables (*N* = 18). The final sample consisted of a total of *N* = 262 participants.

### Sample Size

We performed simulations to estimate the performance of network analyses depending on sample size (100, 250, 350, 500, 750, or 1000 participants), number of variables (5, 10, 15, 20, or 25) and network characteristics (low, medium, or high density) to find the conditions under which we could reasonably expect adequate performance for our analyses. More specifically, we constructed networks for each combination of conditions to serve as ‘true’ networks. We then simulated data based on these network parameters, used network analyses to estimate networks from the simulated data, and compared the estimated networks to the ‘true’ networks. We used four measures of performance, the estimation error (mean absolute difference between estimated and ‘true’ networks), sensitivity (true positives), specificity (true negatives) and the correlation between the simulated and ‘true’ network. The simulations were repeated 100 times for each combination of different sample sizes, numbers of variables and network characteristics.

While we originally aimed to collect data from 500 participants, providing adequate performance for networks with 25 variables (see https://aspredicted.org/8wys-csn4.pdf), we were only able to include ~250 participants. Therefore, we reduced the number of variables included in the main analysis to 15. Averaging over repetitions, the simulations indicated that this should provide great performance for low density networks (error = 0.01, sensitivity = 0.99. specificity = 0.96, correlation = 0.98), good performance for medium density networks (error = 0.02, sensitivity = 0.88, specificity = 0.89, correlation = 0.90) and decent performance for high density networks (error = 0.04, sensitivity = 0.42, specificity = 0.85, correlation = 0.52).

### Study Procedure

Participants were asked to fill in a series of questions and questionnaires online via Qualtrics (Provo, UT). The survey included 27 questions about demographics and pain characteristics, and 20 questionnaires including 104 items in total, which took about 30 minutes to complete. The order of the questionnaires was randomized in blocks so that any potential impact of filling out one questionnaire before/after the other would be balanced out across participants. Participants were allowed to take breaks at any time as long as they completed the study within one week from when they started. At the beginning of the survey, participants gave their informed consent before proceeding to the questions. Ethical permission was obtained from the Ethics Review Committee Psychology and Neuroscience (ERCPN) of Maastricht University.

### Questionnaires

Participants first answered a series of pain-related (e.g., pain onset, duration, location, diagnosis, healthcare visits) and socio-demographic (e.g., age, gender identity, ethnicity, living situation) questions. Then, they completed a series of questionnaires assessing psychological, social, and societal factors related to chronic pain. The selection of these questionnaires was informed by the literature showing that these factors are associated with chronic pain, and aimed to capture a comprehensive range of psychological, social and societal aspects of the chronic pain experience. Additionally, we sought to minimize overlap between constructs and keep the assessment burden manageable for participants.

Traditional and positive psychological instruments were included to assess cognitive, behavioural and emotional aspects known to be associated with chronic pain experiences, such as pain-related worry, anxious and depressive symptoms, pain acceptance, and positive affect (e.g., [36-38]). Reflecting the role of interpersonal relationships and social context, social factors such as social support and pain invalidation were assessed. These variables were selected based on literature showing that social environments can either buffer against or exacerbate pain-related distress, influence treatment outcomes, and affect emotional well-being (e.g., [39-41]). Finally, to capture the broader societal context in which chronic pain occurs, measures of socio-economic position and access to health care were included. These factors were chosen to explore how structural and systemic conditions shape individuals’ experiences of pain, access to support, and overall quality of life (e.g., [42-44]). The questionnaires used in this study, along with their psychometric properties, are presented in Table 1. The questionnaires which were administered but ultimately excluded from the analysis can be found in Table S1.

Five attention checks were embedded throughout the survey to ensure accurate responses from participants. These checks included three direct attention checks (e.g., *Please select "6" to show you are paying attention to this question*), one commitment question (*Do you commit to providing thoughtful answers?*), and one logic question (*Which of the following is a vegetable?*). We also included a honeypot question and CAPTCHA to prevent bots from participating in our study.

### Variables

Reversed items were re-coded prior to calculating the variable values per participant. For most questionnaires and subscales, the items were summed to calculate the value for the variable it measured. There were a few exceptions. For *experiences of discrimination*, we did not include the third item, as this was categorical. For *pain invalidation*, we combined the two subscales into a single variable by adding them up, instead of having separate measures for both subscales. For the Chronic Pain Grade Scale, we calculated the *Characteristic Pain Intensity* and *Disability Scores* following the instructions from the scale developers [62] for our *pain intensity* and *pain disability* variables, respectively. Namely, we took the average of items 1-3 and 5-7, respectively, and multiplied them by 10 to get a score between 0-100. As anxious and depressive symptoms are often strongly correlated, and to reduce the number of variables in our second network analysis, we decided to combine the PHQ-2 and GAD-2 into a single variable reflecting *mood symptoms*, as has been done in previous studies [69,70]. Most variables had good or excellent internal consistency (*Cronbach’s α >* 0.8), while *stress*, *pain intensity* and *positive affect* were acceptable (*α* > 0.7), and *pain acceptance, experiences of discrimination* and *pain avoidance* were somewhat low (*α* > 0.65) in terms of internal consistency. *Pain-means-harm beliefs* had a very low internal consistency (*α* = 0.12), therefore, we decided to conduct our main analyses with the single-item version of the subscale [61]. See Table S2 in the Supplementary Materials for the internal consistency of all variables.

### Translation Procedure

The English survey was translated into Dutch and German to extend data collection to local Dutch and German speaking individuals. Allowing participants to fill out the questionnaire in their native language improves data quality, since this lowers the probability of straight-lining or choosing default answer options [71]. Sociodemographic and pain-related questions were translated by two co-authors of this study. For the questionnaires, we used already validated Dutch and German versions of the scales included in this survey where possible. Eleven questionnaires were available in German and Dutch (see Table S2). For the remaining nine German and nine Dutch questionnaires, a forward–backward translation process was conducted, following the cross-cultural 5-step translation process by Beaton et al. [72]. This translation process aims to ensure that the source and target questionnaires are aligned in terms of meaning, language use and cultural relevance [72]. All translators were recruited through personal networks or announcements on translator apps, participated on voluntary bases and received a small honorarium for their contribution.

In the first step, two native Dutch and two native German speakers fluent in English independently translated the questionnaires. Two of the authors acted as forward translators, one in each language. In step 2, the translators compared their versions and discussed discrepancies until they reached a consensus on a single version per language. Next, two native English-speaking back-translators that were proficient in Dutch or German, translated the items back to English. Discrepancies between the backtranslations as well as the backtranslations and original items were documented. All translators and an additional two chronic pain experts per language evaluated the translations for accuracy, clarity and fidelity through an online survey in Qualtrics (Provo, UT). In the fourth step, all translators and chronic pain experts were invited to a meeting (one per language) to discuss the items with the most divergent quality ratings or biggest discrepancies in the backtranslations. The original translations were adjusted based on these discussions. Since we used data collected in multiple languages in one analysis, we also evaluated the existing translations and made a few minor adjustments to ensure the English, Dutch and German translations were as similar as possible. In the last step, these pre-final versions were piloted with four individuals per language who experience chronic pain to evaluate clarity, relevance, and comprehensibility. Their feedback was incorporated to improve the final versions, ensuring cultural appropriateness and content validity (Beaton et al., 2000). The final translations, and the minor adjustments we made to existing translations can be requested from the corresponding author. In the study sample, *N* = 184 (70.2%) participants filled the survey out in English, *N* = 55 (21.0%) in German, and *N* = 23 (8.8%) participants in Dutch.

### Data Analysis

#### Model assumptions.

We checked for violations of normality using histograms and Q-Q plots, as well as the multivariate normality test from the *mvn* function in the MVN package [73]. None of the variables had strictly normal distributions, however, only five showed a substantial violation (skewness > ±0.5; i.e., *emotional support, depressive symptoms, pain invalidation, pain-related worry* and *experiences of discrimination*). Following recommendations based on an extensive simulation study [74], we applied a non-paranormal transformation to the data to maintain high expected model performance (precision, specificity, correlation and sensitivity to top 50% of edges > .8). This greatly improved data normality, and Henze-Zirkler’s test for multivariate normality was now non-significant for both the replication (test statistic = 0.96, p = .27) and the extended (test statistic = 1.00, p = .11) networks, though *emotional support* and *experiences of discrimination* still showed substantial skewness (−0.5 and 0.4, respectively, see Figures S1 and S3). We created scatter plots for each combination of variables to check for linearity, and identified no signs of violations of this assumption, apart from the ceiling and floor effects in *emotional support* and *experiences of discrimination* being visible (see Figures S2 and S4).

#### Network analysis.

**Figure 1.**
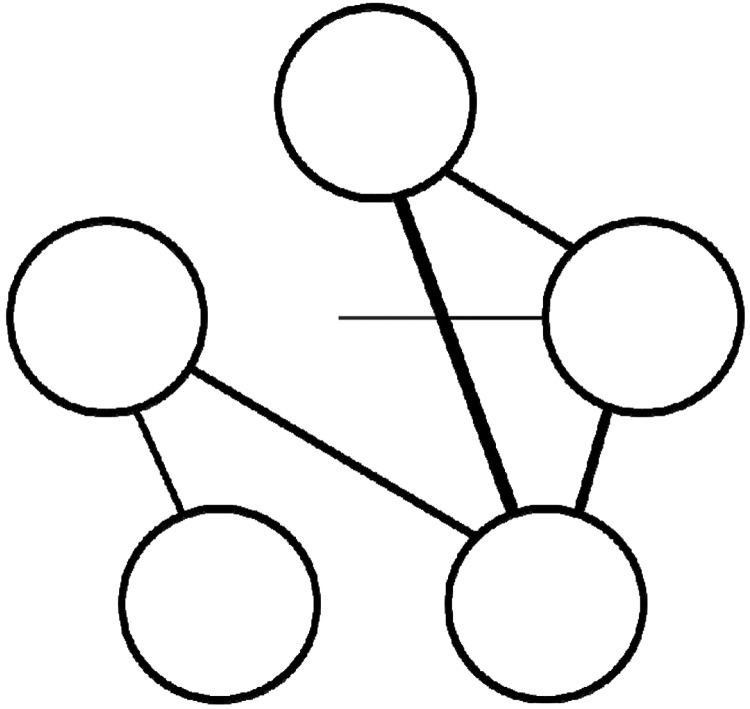
Illustration of a network model in which each variable is presented as a circle (nodes), while the lines between them (edges) represent the partial correlations between each pair of variables. Thicker lines indicate stronger partial correlations.

We used Gaussian Graphical Models (GGMs) to explore the relationships between variables in our study. These models estimate partial correlations between each combination of variables while correcting for all others. As such, the results indicate the unique, direct relationships between all variables. This can be represented as a network, in which each variable is a circle (node), while each partial correlation is a line between nodes (edge; see Figure 1). Thicker lines indicate stronger partial correlations. We used the *estimateNetwork* function from the *bootnet* package in R with default settings for model estimation [75,76]. For this analysis, a series of linear multiple regressions is performed (one for each variable) in combination with least absolute shrinkage and selection operator (LASSO) regularization using 10-fold cross-validation for parameter selection. This regularization protects against issues with multicollinearity and reduces overfitting, which is especially helpful in situations where there are many variables. More specifically, it looks for the smallest combination of predictors that can still accurately predict the outcome by adding a penalty for more complex models and decreasing regression coefficients towards zero. We controlled for false positives by applying thresholding to estimates following Loh and Wainwright [77].

We estimated two partial correlation networks for our main analyses. First, we included six variables covered in two previous large-scale network studies of chronic pain, namely *pain intensity, pain disability, anxious symptoms, depressive symptoms, pain-means-harm beliefs* (named pain-related fear in other studies) and *pain avoidance*. Then, we expanded the model to include the 15 main variables in our study: *pain intensity, pain disability, quality of life, pain-related worry, pain-means-harm beliefs, pain avoidance, pain acceptance, positive affect, mood symptoms, stress, emotional support, pain invalidation, experiences of discrimination, access to healthcare*, and *financial worry*.

#### Network stability and predictability.

In line with Epskamp et al. [75], we examined the stability of the estimated networks using bootstrapping. Specifically, we repeated network estimation 5000 times with bootstrapped samples, which means that, for each sample, participants were randomly selected from the full sample with replacement. The percentage of bootstrapped samples in which an edge is identified, and the distribution of the edge estimates across bootstrapped samples provide an indication of the stability and accuracy of the edges in the main analysis, with higher percentages and narrower distributions pointing to more stable and accurate partial correlations. We also estimated the predictability of the nodes [78]. This measure represents the proportion of variance in one variable explained by all other variables (*R^2^*).

### Software and Scripts

All data cleaning and analysis was done in R Statistical Software (v4.4.1; R Core Team, 2024). The main packages we used include *mgm* (v1.2.13; [76]), *bootnet* (v1.5; [75]) and *qgraph* (v1.9.3; [79]). The scripts used in this study are available through OSF (https://osf.io/p3djf/?view_only=8f243e0b6eb5481087e4fb12c56f9e70).

### Positionality Statement

Conducting research involves making many decisions along the way. Positionality statements inform readers about the background and experiences of authors that shape the lens through which they engaged with the presented research. While positionality statements are a longstanding practice in qualitative research [80], there have been increasing calls for quantitative researchers to reflect and report on their positionality as well [81-83]. In line with these calls, we offer the following positionality statement.

We all grew up and live in Western countries and have been trained in the Eurocentric scientific tradition. While some of us have some insight into other perspectives, either through personal ties or engaging with the work of non-Western scholars, we believe the presented work largely represents Western views on science, pain and health. Most of us have a background in psychology, with one having clinical training and expertise. One author has a background in cultural anthropology, and we aimed to incorporate anthropological and sociological perspectives into this project, however, psychological perspectives provided the main guidance and direction. Five of us have lived experiences of chronic health conditions, including chronic pain symptoms. Our team consists of six women and one man. Five of us are White Europeans, one of us a Black American, and one of us from the Caucasian mountain area, now living in Western Europe. While we thus have some relevant experiences to health and other societal inequities, we also carry many privileges (e.g., financial stability, reasonable health literacy) and do not by far match the diverse backgrounds and experiences of our sample.

## Results

### Sample Characteristics

The demographic and pain characteristics of our sample are presented in Table 1. Overall, the sample was fairly young (M = 31.1, SD = 15.2), and consisted primarily of women (78.6%) of European ethnicity (85.1%) who have (38.9%) and/or are currently working towards (52.7%) a university degree. On average, the sample had moderate pain intensity (M = 54.7, SD = 16.2) and pain disability (M = 48.6, SD = 23.8), though the majority of the sample had pain for over 2 years (87.2%). Participants often reported more than one pain location, with the most common pain locations being the face, head or neck (48.9%) and the feet, hips, knees or legs (51.1%).

### Replication Network

**Figure 2.**
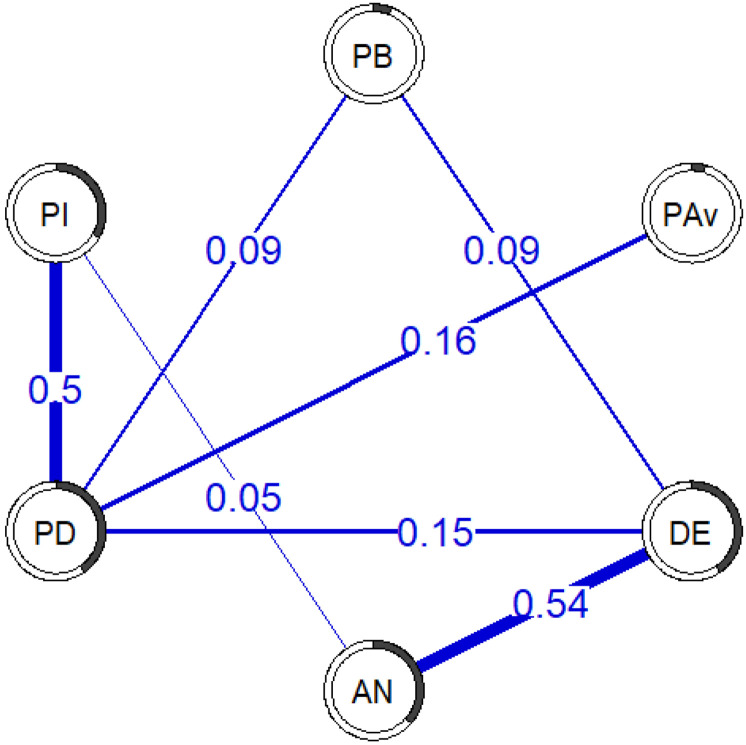
Cross-sectional replication network. The lines between variables (edges) represent partial correlations, with thicker lines indicating stronger partial correlations. The pie charts around each circle (node) represent the proportion of variance explained by the other variables (*R*^*2*^). PB = Pain-means-harm beliefs, PAv = Pain avoidance, DE = Depressive symptoms, AN = Anxious symptoms, PD = Pain disability, PI – Pain intensity

The partial correlation network estimated from similar variables as two large sample studies [28,29] is presented in Figure 2. Seven out of the 15 possible edges were estimated to be present and all of them were positive, with partial correlations ranging from .05 (*anxious symptoms - pain intensity*) to .54 (*depressive symptoms - anxious symptoms;* see Table S3 for an overview of all partial correlations). The bootstrapping procedure showed that all estimated edges were present in the majority of bootstrapped samples, with the four strongest edges present more than 97% of the time (see Figure S5). Furthermore, the 95% quantiles around the edges were .17-.32 wide, indicating that, in 95% of the bootstrapped samples, the estimated partial correlations fell within a ±.08-.16 window of the estimates in the main analysis of the full sample. The predictability varied from node to node, with the most variance explained for *depressive symptoms* (*R*^*2*^ = .40 or 40%), and least amount of variance explained for *pain avoidance* (*R*^*2*^ = .05 or 5%).

### Extended Network

**Figure 3.**
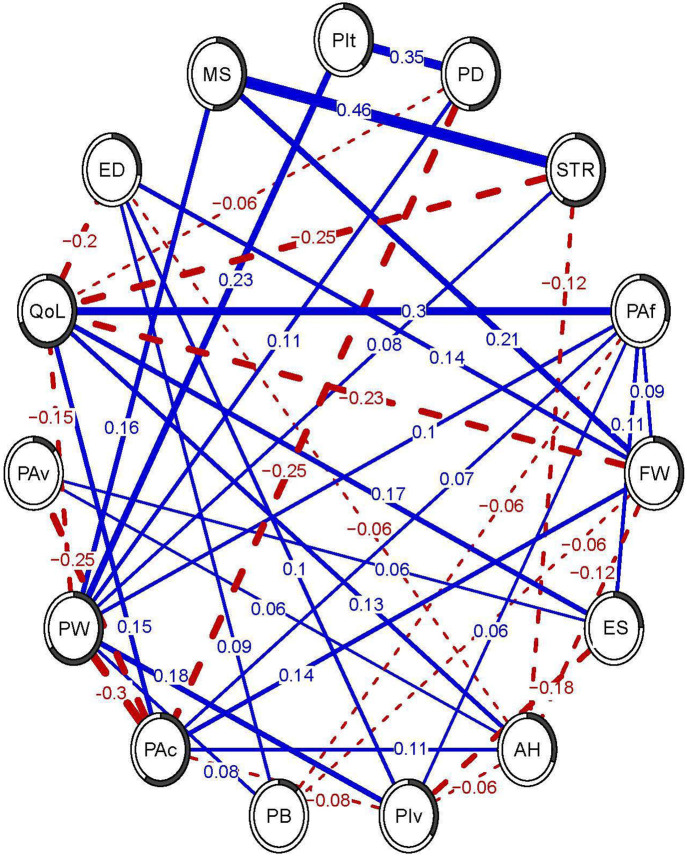
Cross-sectional extended network. The lines between variables (edges) represent partial correlations, with blue solid lines indicating positive relationships, red dashed lines indicating negative relationships, and thicker lines indicating stronger partial correlations. The pie charts around each circle (node) represent the proportion of variance explained by the other variables (*R*^*2*^). PIt = pain intensity, PD = pain disability, STR = stress, PAf = positive affect, FW = financial worries, ES = emotional support, AH = access to health care, PIv = pain invalidation, PB = pain-means-harm belief, PAc = pain acceptance, PW = pain-related worries, PAv = pain avoidance, QoL = quality of life, ED = experiences of discrimination, MS = mood symptoms

The partial correlation network estimated from the extended set of variables is presented in Figure 3. Out of the 105 possible edges, 41 were estimated to be present. Twenty-four of the edges were positive, with the largest partial correlation existing between *mood symptoms* and *stress* (*r* = .46). Sixteen of the edges were negative, with the largest partial correlation existing between *pain-related worry* and *pain acceptance* (*r* = −.30). See Table S4 for an overview of all partial correlations. Among our pain outcomes of interest, *pain intensity* had direct relationships with *pain-related worry* (*r* = .23) and *pain disability* (*r* = .35), and *pain disability* had additional direct relationships with *pain acceptance* (*r* = −.25), *pain-related worry* (*r* = .11), and *quality of life* (*r* = −.06). *Quality of life* had relationships with nine of the other variables in the network, among which *positive affect* (*r* = .30), *stress* (*r* = .−25), *financial worry* (*r* = −.23) and *experiences of discrimination* (*r* = −.20) showed the strongest associations. The bootstrapping procedure showed that all estimated edges were present in the majority of bootstrapped samples, and all edges estimated at *r* = ±.1 were present at least 75% of the time (see Figures S6 and S7). In addition, the 95% quantiles around the edges were .08-.32 wide, indicating that in 95% of the bootstrapped samples, the estimated partial correlations fell within a ±.04-.16 window of the estimates in the analysis of the full sample. The predictability varied from node to node, with the most variance explained for *quality of life* (*R*^*2*^ = .69 or 69%), and least amount of variance explained for *pain beliefs* (*R*^*2*^ = .11 or 11%).

## Discussion

In this study, we aimed to replicate previous networks of psychological factors related to chronic pain disability, and build on this work by including social and societal factors in our extended network analysis. Despite using different measures and recruiting directly from the community instead of a specialized pain clinic, our replication network showed many similarities to previously published networks [28,29], including pain disability being related to pain intensity, depressive symptoms, pain avoidance and pain-means-harm beliefs. In our extended network, pain intensity and disability were still strongly related to each other, but now only showed direct relationships with pain-related worry and pain acceptance. Pain avoidance, pain-means-harm beliefs and depression (mood symptoms) were still indirectly related to pain disability and pain intensity, together with pain invalidation, financial worries, access to health care, stress and positive affect. Quality of life was directly related to nine of the fourteen variables, most notably positive affect, stress, financial worry and experiences of discrimination.

Our sample was recruited directly from the community, e.g., from public spaces and primary health care clinics, and included people with a range of pain locations and severity (e.g., pain duration, intensity, disability). Even though the two large-sample studies we aimed to replicate included samples recruited from specialized pain clinics [28,29], our findings were similar. While our network had fewer connections overall, the strongest relationships were all replicated, with one notable exception. Namely, no relationship was identified between pain-means-harm beliefs and pain avoidance. This likely occurred because we used different measures. Notably, we measured pain avoidance through items asking how often participants avoid activities they associate with pain, whereas van der Wijk et al. [29] examined beliefs on the importance of avoiding activities to prevent further harm (Activity Avoidance subscale Tampa Scale of Kinesophobia; [84]), and Zhao et al. [28] used a combined fear-avoidance measure (full TSK). Even so, both pain avoidance and pain-means-harm beliefs were related to pain disability, as in previous studies [27-29,85,86]. Similarly, the relationships of pain disability with both pain intensity and depressive symptoms we found have commonly been observed by others as well [27-29,86]. Overall, we found a similar network of psychological factors related to chronic pain, indicating that pain disability has unique relationships with almost all of them.

In the extended network analysis, pain disability and pain intensity were only related to each other and to pain acceptance and pain-related worry, apart from a weak relationship between pain disability and quality of life. In line with psychological research on chronic pain, our analysis thus highlighted core concepts from two distinct psychological models of chronic pain (i.e., the psychological flexibility model and fear-avoidance model; [9,16]) as being the most directly related to pain disability and pain intensity. This fits well with the idea that positive psychological factors can complement earlier psychological models of pain [87]. While pain avoidance, pain-means-harm beliefs and mood symptoms were not directly related to pain disability, as in the replication network, their relationships with pain acceptance and pain-related worry suggest a potential indirect relationship with pain disability. A similar indirect relationship between fear-avoidance and pain disability through pain acceptance was also observed by Ackerblom et al. [27]. In addition, pain-related worry and pain acceptance were associated with pain invalidation, stress, financial worry, positive affect and access to health care, suggesting that several social-societal factors may also be related to pain disability through these psychological coping strategies. Overall, this pattern of results suggests that pain acceptance and pain-related worry play a primary role in understanding pain disability, while other psychological and social-societal factors play an indirect role.

Indeed, this pattern of relationships invites us to look beyond the immediate psychological factors to what may shape individual differences in these factors. Our results indicate that invalidating responses from both health care providers and immediate others are associated with more pain-related worry and less pain acceptance. Although our cross-sectional design leaves the direction of influence open, several qualitative studies have highlighted a lack of understanding and support from others as a barrier to pain acceptance [88-90], suggesting a causal effect. A systematic meta-synthesis mapping the consequences of pain invalidation more broadly also identified increased worry and self-doubt as an important outcome [91]. While we know of no studies examining the relationship of pain acceptance with access to health care and financial worry, theoretical models of pain and more general health inequities have proposed that lower socio-economic position exerts its negative effect through shaping coping strategies [8,92-94]. Our findings align with this idea that responses to chronic pain, such as worry and acceptance, are impacted by the circumstances people live in. Since improving pain coping strategies is often the primary aim of non-pharmacological treatment for chronic pain, one may wonder to what extent such an individual focus can be effective when there are external factors that contribute to the use of these coping strategies in the first place, as others have done before us (e.g., [95]).

The importance of social and societal factors in the context of chronic pain is further emphasized by the substantial direct relationships of quality of life with stress, financial worries, experiences of discrimination, emotional support and access to health care. While this may not be surprising, it is notable that, among our sample of people living with chronic pain, these factors had stronger relationships to quality of life than pain disability and pain intensity. Furthermore, it is noteworthy that these relationships appeared despite having a relatively well-resourced and connected sample (e.g., high social support, low financial worry). We would expect the strength of such relationships to be even more pronounced in a sample including people from a wider range of backgrounds, for example in terms of socio-economic position and race.

Since quality of life is often an important outcome of treatment (e.g., [96,97]), our findings suggest that taking social and societal factors into account during treatment alongside more typically targeted psychological factors could lead to more effective interventions, a point also made by others [8,98-100]. Ashton-James et al. [26] recently highlighted the importance of social factors in chronic pain treatment as well and provide some suggestions on how to move towards incorporating them in clinical practice. Although societal factors are more difficult to address in individual interventions, several models have been developed to highlight health care practices that may reduce the negative impacts of societal stressors for patients undergoing treatment (e.g., RESTORATIVE model, [101]; EQUIP Healthcare model, [102]). A study implementing the EQUIP Healthcare model in four primary care clinics found that higher levels of equity-oriented care was associated with better health outcomes [103]. Interestingly, this association was mediated by patient confidence in managing their health, highlighting a potential psychological mechanism, similar to our findings.

Naturally, the impact of social and societal factors cannot be mitigated by adjustments at the individual level alone. Indeed, it is likely that addressing their root causes would be even more effective, as others have put forward as well (e.g., [104]). For example, Hood et al. [101] emphasize that the most impactful actions for addressing race-related pain inequities require structural changes from health care providers and the institutions they work at to prevent racialized people from being exposed to discrimination. Alongside our cross-sectional findings, longitudinal findings that more financial worry was associated with higher pain ratings on a daily level, suggest that alleviating economic hardship could also reduce chronic pain [105]. While this is challenging, long-term work, several initiatives have already been taken to start addressing societal causes of pain inequities. For example, Hirsch et al. [106] developed and tested a virtual intervention to reduce treatment bias related to race and socio-economic position in health care providers. Similarly, training for health care providers has been developed to improve empathic validation when communicating with people experiencing pain [107,108]. Though not specific to chronic pain, the need for policy and political interventions to address health inequities has been emphasized, for example by improving access to affordable care, housing and education [109].

More work on the interactions between social-societal and psychological factors is needed to illuminate the direction and nuances of the interactions between psychological, social and societal factors. While evidence for differences in pain outcomes related to sociodemographic factors has accumulated, we know of little work that has examined the mechanisms through which societal inequities lead to such diverse outcomes. Our study provides preliminary insights by highlighting how specific social-societal and psychological factors interact, pointing to potential mechanisms. Since our survey method provided only surface level information on a single point in time, much remains unknown about the direction and meaning of the effects we found. For example, we were surprised that more financial worry was associated with more pain acceptance in our sample, but since our online survey method does not allow for in-depth understanding, it is difficult to interpret this finding. Seeing the complex and multifactorial nature of chronic pain, we agree with previous work [8,101] that interdisciplinary collaborations across the fields of public health, medical anthropology, psychology and sociology could provide the insights needed to guide future efforts.

Our study has several limitations. While our sample size was adequate, as indicated by both a-priori simulations and post-hoc stability measures, the accuracy of our network estimates was somewhat lower than in previous studies with larger samples. We managed to recruit a community sample diverse in their pain characteristics; however, it was considerably skewed in sociodemographic characteristics, most notably consisting largely of young white women with or on track to have university-level education. In addition, our recruitment and data collection methods may have encouraged participation from those who are most interested in sharing their experiences with pain, and may have discouraged those who have fewer digital skills and/or do not speak English. As such, our sample is not representative of all people experiencing chronic pain and therefore our results may not generalize to the whole population. Our use of a one-time online survey further limited our ability to make causal inferences. Furthermore, our results may be influenced by socially desirable answers and differences in the environment in which participants filled out the questionnaires. We selected short forms where possible to reduce the participant burden, however, these may have provided a limited estimate of the factors of interest. This is especially likely for the pain-means-harm variable, as we ended up using the 1-item version due to low internal consistency for the 2-item version in our sample. Even so, the questionnaire was relatively long, which may have led to motivation and attention dropping towards the end. The items for pain avoidance were accidentally measured on a 4-point instead of a 5-point scale, deviating from the original Activity Patterns Scale [48]. Lastly, a substantial proportion of our sample filled out the questionnaire in a language that is not their mother tongue, which may have impacted their answers.

Overall, our results emphasize that, when taking important psychological factors into account, social and societal remain directly and indirectly related to important pain outcomes. Specifically, our results showed that psychological, social and societal factors all directly relate to quality of life among people with chronic pain, with stress, financial worry, positive affect and experiences of discrimination being the most prominent. Pain disability and pain intensity only had direct relationships with pain-related worry and pain acceptance, however, these factors were in turn related to social and societal factors, suggesting that the context people live in can act as a barrier or facilitator for better pain management. Future work should examine the interactions between psychological and social-societal factors in more depth to understand how they shape pain coping responses and can thus be best approached through intervention. In addition, our findings emphasize the need for structural changes (e.g., addressing discrimination, pain invalidation and socio-economic inequities) to alleviate the burden of chronic pain. Considering the limitations, we note that the results should be taken as a preliminary step that may inspire future work.

## Supplementary Material

Supplement 1

## Figures and Tables

**Table 1:** Summary of Study Variables, Questionnaires, and their Psychometric Properties

Variable	Questionnaire	No of Items	Answer scale	Validity and reliability	References
Access to Health Care	Subscales Accessibility and Convenience of the Patient Satisfaction Questionnaire Short Form (PSQ-18)	4	1 (strongly agree) to 5 (strongly disagree)	α = .75 adequate construct validity	Marshall & Hays (1994) [45]
Anxious Symptoms	General Anxiety Disorder 2-item scale (GAD-2)	2	0 (not at all) to 3 (nearly everyday)	α = .69 r =.69 good construct validity	Hitchon et al., 2020; Plummer et al., 2016 [46,47]
Coping Strategies	Subscales pain avoidance, excessive persistence, pacing to conserve energy for valued activities of the Activity Patterns Scale (APS)	9 (3 per subscale)^a^	0 (not at all) to 4 (always)^b^	α = .67-.91 r = .72-.82 good construct validity	Esteve et al., (2016), Hotz-Boendermaker et al. (2024) [48,49]
Depressive Symptoms	Patient-Health Questionnaire-2 (PHQ-2)	2	0 (not at all) to 3 (nearly everyday)	α = 0.76–.83 r = .70–.92 good convergent, construct and criterion validity	Gelaye et al. (2016), Kroenke et al. (2003), Löwe et al. (2005), Maroufizadeh et al. (2019), Yu et al. (2011) [50-54]
Emotional Support	PROMIS 4-item Emotional Support Scale	4	1 (never) to 5 (always)	α = .90–.93 good criterion and construct validity	Cella et al. (2010), Hahn et al. (2014) [55,56]
Experiences of Discrimination	Everyday Discrimination Scale short form	3	First 2 items: 1 (almost everyday) to 6 (never) Third item: asks about main reasons for these experiences, if relevant.	α = .77 r=.69	Benner et al., 2022 Krieger et al., 2005 [57,58]
Financial Worry	Financial Anxiety Scale (FAS-2)	2	1 (never) to 7 (always)	α = .94 good construct validity	Archuleta et al. (2013) [59]
Pain Acceptance	Chronic Pain Acceptance Questionnaires (CPAQ-2)	2	0 (never true) to 6 (always true)	accounted for 61% of variance in the 20-item scale (p < .001)	Vowles et al., 2020 [60]
Pain-Means-Harm Beliefs	2-item version of the harm subscale of the Survey of Pain Attitudes (SOPA)	2	0 (very untrue) to 4 (very true)	strong association (r = .81-.83) with the long form of the subscale original SOPA: α = .71 - .81 r= .63 - .68	Jensen et al., 2003 [61]
Pain Intensity and Disability	Chronic Pain Grade Scale (CPGS)	7 (3 items for pain intensity, 4 items for pain disability)	Pain Intensity: 0 (no pain) to 10 (pain as bad as could be) Disability: 0 (e.g., no interference/no change) to 10 (e.g., extreme interference/extreme change)	α=.67-.9132 high criterion and construct validity (including good convergent and discriminant validity)	Von Korff et al. (1992), Smith et al. (1997) [62,63]
Pain Invalidation	Subscales invalidation by immediate others and invalidation by health care providers of the Pain Invalidation Scale	12	1 (strongly disagree) to 7 (strongly agree)	By immediate others: α =.92, r =.75 By health care providers: α =.93,r =.69	Nicola et al., 2022 [64]
Pain-related Worry	Concerns About Pain (CAP) Scale	6	1 (never) to 5 (always)	r=.70 strong construct validity	Amtmann et al., 2020 [65]
Stress	Perceived Stress Scale (4 item version)	4	0 (never) to 4 (very often)	α =.74 Spearman-Brown split-half reliability = .76	Vallejo et al., 2018 [66]
Positive Affect	Positive affect subscale of the short form of the Positive and Negative Affect Schedule (PANAS)	5	1 (never) to 5 (always)	α=.78 r =.84	Thompson et al., 2007 [67]
Quality of Life	EUROHIS-QOL	8	1 (not at all) to 5 (completely)	α = .83 good structural, convergent, and discriminant validity	Schmidt et al. (2006) [68]

*Note.* This table presents the internal consistency (α) and test–retest reliability (r) of the questionnaires in previous studies.

a Only the three items from the pain avoidance subscale were used in the main analyses

b By accident, the current questionnaire was set up with 4 instead of 5 answer options for this scale

**Table 2:** Demographic and Pain Characteristics of the Sample

Demographic characteristics		Pain characteristics	
Age*	31.1 ± 15.2 (18-86)	Pain intensity	54.7 ± 16.2 (10-93)
Biological sex		Pain disability	48.6 ± 23.8 (0-100)
□ Female	221 (84.7%)		
□ Male	38 (14.6%)	Pain onset	
□ Other or prefer not to answer	3 (0.8%)	□ Gradual	129 (49.2%)
Gender identity		□ Sudden, with clear reason	50 (19.1%)
□ Man	40 (15.3%)		
□ Non-binary	10 (3.8%)	□ Sudden, without clear reason	73 (27.9%)
□ Woman	206 (78.6%)		
□ Other or prefer not to answer	6 (2.3%)	□ Other	10 (3.8%)
Racial/ethnic background*		Pain duration	
□ African	6 (2.3%)	□ 3-6 months	21 (8.0%)
□ East, South and Southeast Asian	5 (1.9%)	□ 6 months - 1 year	16 (6.1%)
□ European	223 (85.1%)	□ 1-2 years	20 (7.6%)
□ Latin American	1 (0.4%)	□ 2-5 years	75 (28.6%)
□ Middle Eastern and Central Asian	12 (4.6%)	□ More than 5 years	130 (49.6%)
□ Mixed European & non-European	8 (3.1%)	Pain location	
□ North American or Australasian	6 (2.3%)	□ Arms/hands/shoulders	115 (43.9%)
Minority status			
□ No	230 (87.8%)	□ Back	118 (45.0%)
□ Yes	32 (12.2%)	□ Chest	43 (16.4%)
Living situation*		□ Digestive system	67 (25.6%)
□ Living alone	87 (33.2%)	□ Face/head/neck	128 (48.9%)
□ Living with partner and/or kids	72 (27.5%)	□ Feet/hips/knees/legs	134 (51.1%)
□ Living with parents/guardians	41 (15.6%)		
□ Living with roommates	61 (23.3%)	□ Genital	22 (8.4%)
Living environment		□ Widespread	48 (18.3%)
□ Village	22 (8.4%)	□ Other	33 (12.6%)
□ Small town	43 (16.4%)	Health care visits	
□ Middle-sized town	74 (28.2%)	□ No	13 (5.0%)
□ Big city	99 (37.8%)	□ Yes, GP/physiotherapist only	52 (19.8%)
□ Metropolis	24 (9.2%)		
Education		□ Yes, other specialist(s)	197 (75.2%)
□ Primary education	2 (0.8%)		
□ Secondary education	122 (46.6%)	Pain diagnosis	
□ Post-secondary education	36 (13.7%)	□ No	86 (32.8%)
□ Bachelor's degree or equivalent	56 (21.4%)	□ Yes	176 (67.2%)
□ Master's degree or equivalent	46 (17.6%)	Current treatment*	
Employment		□ No	150 (57.5%)
□ Disability benefits	24 (9.2%)	□ Yes	111 (42.5%)
□ Retired	16 (6.1%)	Other diagnoses	
□ Studying	105 (40.1%)	□ No	125 (47.7%)
□ Studying & working (paid & unpaid)	33 (12.6%)	□ Yes	137 (52.3%)
□ Unemployed	10 (3.8%)		
□ Unpaid work	3 (1.1%)		
□ Working full-time	37 (14.1%)		
□ Working part-time	34 (13.0%)		

* These variables had 1 missing value each

## Data Availability

The data used in this study is available on OSF: https://osf.io/p3djf/?view_only=8f243e0b6eb5481087e4fb12c56f9e70.
