# Supplementary material for: Social and Societal Factors Interact with Psychological Factors to Shape Pain Outcomes in a Community Sample with Chronic Pain: A Network Study
Source: medRxiv. 2025 Sep 3:2025.08.29.25334446. Preprint. [Version 1] doi: 10.1101/2025.08.29.25334446 (PMC12424911; doi:10.1101/2025.08.29.25334446)
Supplement: Supplement 1 [file media-1.docx]

###### **Supplementary materials**

1. **Questionnaires not selected for data analysis**

**Table S1**

*Summary of Excluded Variables, Questionnaires, and their Psychometric Properties*

| **Variable** | **Questionnaire** | **No of Items** | **Answer scale** | **Validity and reliability** | **For further details** |
| --- | --- | --- | --- | --- | --- |
| Internalised Stigma | Alienation subscale of Internalised Stigma in Chronic Pain Scale | 6 | 1 (strongly disagree) to 4 (strongly agree) | α=.90 | Waugh et al. (2014) |
| Optimism | Positively worded items of the Revised Life Orientation Test (LOT-R)* | 3 | 0 (strongly disagree) to 4 (strongly agree) | α = .71  r = .79 | Herzberg et al. (2006), Scheier et al. (1994) |
| Pain Resilience | Pain Resilience Scale (PRS) | 12 | 0 (not at all) to 4 (all the time) | α = .93  r = .80  acceptable convergent and external validity | Ankawi et al. (2017), Slepian et al. (2016) |
| Patient-Doctor Relationship | Patient-Doctor Relationship Questionnaire (PDRQ-9) | 9 | 1 (not at all appropriate to 5 (totally appropriate) | α = .94-.96  r = .61  good convergent and discriminant validity | Van der Feltz-Cornelis et al. (2004), Zenger et al. (2014), Porcerelli et al. (2014) |
| Trauma | Adverse Childhood Experiences Questionnaire for Adults – Short Form (ACE-Q-2) | 2 | Checkboxes (either applies or not) | α = .76–.83^a^  r = .71–.913^a^ | Irshad and Lone (2025), Schauss et al. (2021), Wade et al. (2017), Zanotti et al. (2018) |

*Note.* This table presents the internal consistency (α) and test–retest reliability (r) of the questionnaires in previous studies.

*^a^ Based on ACE-10 reliability data, as data for the ACE-Q-2 are limited*

## **Available translations**

**Table S2**

*Overview of Available Translations Used in this Study*

| **Questionnaire** | **German version** | **Dutch version** |
| --- | --- | --- |
| Chronic Pain Grade Scale | Klasen et al., 2004 [1] | NA – translated by authors |
| Perceived Stress Scale - 4 |  | Longitudinal Aging Study Amsterdam [https://lasa-vu.nl/wp-content/uploads/2021/ 03/LASA104_quest_nl.pdf](https://lasa-vu.nl/wp-content/uploads/2021/03/LASA104_quest_nl.pdf) |
| Revised Life Orientation Test (LOT-R) | Glaesmerl et al., 2008 [2] | Klooster et al., 2010 [3] |
| Positive and Negative Affect Schedule (PANAS) – short form | Röcke & Grühn, 2003 [4] | Engelen et al., 2006 [5] |
| Patient-Health Questionnaire-2 (PHQ-2) | Löwe et al., 2010 [6] |  |
| Adverse Childhood Experiences Questionnaire for Adults – Short Form (ACE-Q-2) | Wingenfeld et al., 2010 [7] | NA – translated by authors |
| General Anxiety Disorder 2-item scale (GAD-2) | Löwe et al., 2008 [8] | ECFS Mental Health Working Group [https://www.ecfs.eu/sites/default/files/ general-content-files/working-groups/Mental%20Health/GAD7 _Dutch%20for%20Belgium.pdf](https://www.ecfs.eu/sites/default/files/general-content-files/working-groups/Mental%20Health/GAD7_Dutch%20for%20Belgium.pdf) |
| Patient-Doctor Relationship Questionnaire (PDRQ-9) | Zenger et al., 2014 [9] | van der Feltz-Cornelis et al., 2004 [10] |
| Chronic Pain Acceptance Questionnaires (CPAQ-2) | Nilges et al., 2007 [11] | Trompetter et al., 2011 [12] |
| Activity Patterns Scale | Hotz-Boendermaker et al., 2024 [13] | Received from authors of Esteve et al., 2016 [14] |
| EUROHIS-QOL | Brähler et al., 2007 [15] | De Vries & Van Heck, 1996 [16] |
| PROMIS 4-item Emotional Support Scale | NA – see author translations below | PROMIS Health Organization [https://drive.google.com/file/d/ 1PWJD7op4rXlt261VhLKp09 qszhBXkG10/view?usp=drive_link](https://drive.google.com/file/d/1PWJD7op4rXlt261VhLKp09qszhBXkG10/view?usp=drive_link) |
| Patient Satisfaction Questionnaire | NA – translated by authors | Quakunde ([https://www.quantitativeskills.com/ quakunde/PSQ18NL.pdf](https://www.quantitativeskills.com/quakunde/PSQ18NL.pdf)) |

1. **Internal consistency**

**Table S3**

*Internal Consistency of Variables Used in Analyses*

| **Variable** | **Cronbach’s alpha** |
| --- | --- |
| *Access to Health Care* | 0.80 |
| *Anxious symptoms* | 0.84 |
| *Depressive symptoms* | 0.81 |
| *Emotional Support* | 0.94 |
| *Experiences of Discrimination* | 0.69 |
| *Financial Worry* | 0.89 |
| *Pain Acceptance* | 0.67 |
| *Pain Avoidance* | 0.67 |
| *Pain Beliefs* | 0.12 |
| *Pain Disability* | 0.88 |
| *Pain Intensity* | 0.73 |
| *Pain Invalidation* | 0.92 |
| *Pain-related worry* | 0.89 |
| *Mood symptoms* | 0.84 |
| *Stress* | 0.79 |
| *Positive Affect* | 0.76 |
| *Quality of Life* | 0.83 |

1. **Model assumptions**


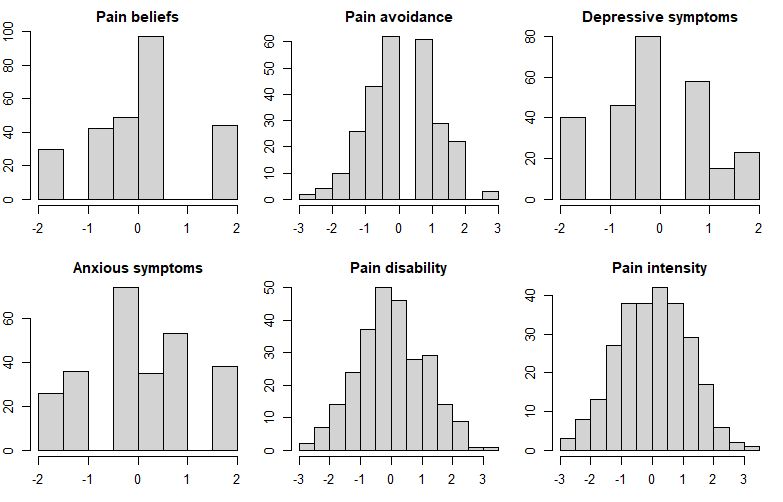


**Figure S1.** Histograms of variables used in replication network analysis after non-paranormal transformation.


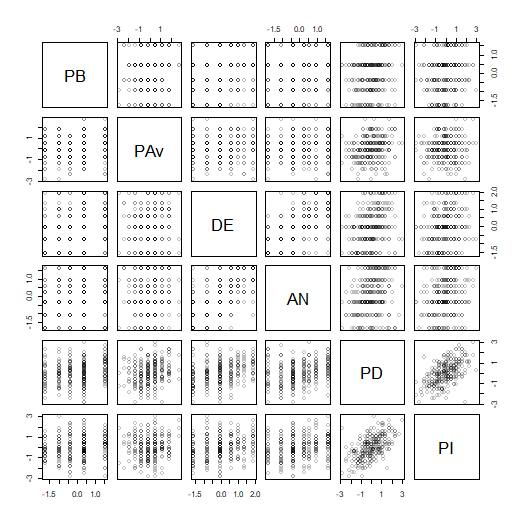


**Figure S2.** Pair-wise scatter plots of variables used in replication network analysis after non-paranormal transformation. PB = Pain-means-harm beliefs, PAv = Pain avoidance, DE = Depressive symptoms, AN = Anxious symptoms, PD = Pain disability, PI – Pain intensity


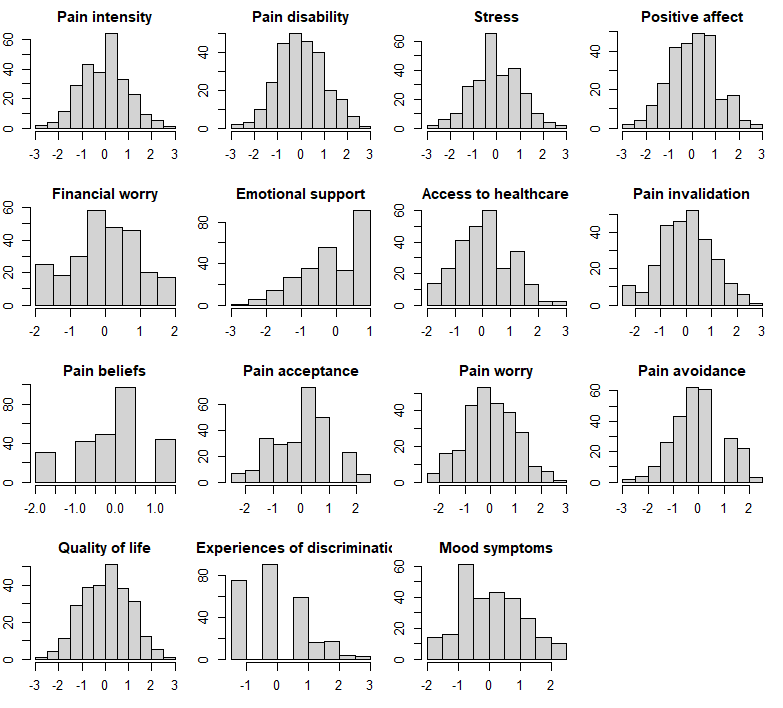


**Figure S3.** Histograms of variables used in extended network analysis after non-paranormal transformation.


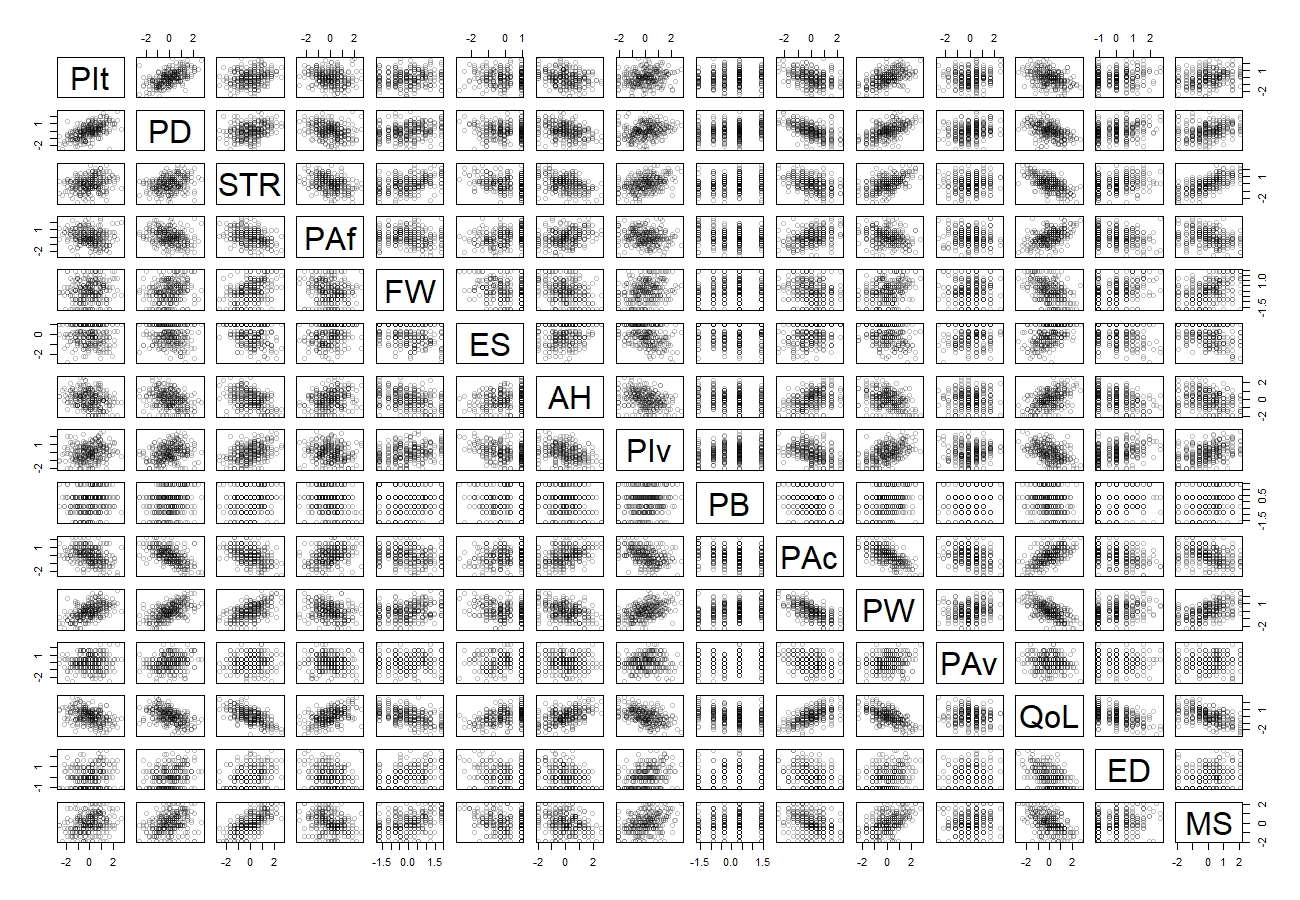


**Figure S4.** Pair-wise scatter plots of variables used in extended network analysis after non-paranormal transformation. PIt = pain intensity, PD = pain disability, STR = stress, PAf = positive affect, FW = financial worries, ES = emotional support, AH = access to health care, PIv = pain invalidation, PB = pain-means-harm belief, PAc = pain acceptance, PW = pain-related worries, PAv = pain avoidance, QoL = quality of life, ED = experiences of discrimination, MS = mood symptoms

1. **Partial correlation tables for replication and extended networks**

**Table S4**

*Partial Correlations in Replication Network*

|  | **PB** | **PAv** | **DE** | **AN** | **PD** | **PI** |
| --- | --- | --- | --- | --- | --- | --- |
| **PB** | . | . | .09 | . | .09 | . |
| **PAv** | . | . | . | . | .16 | . |
| **DE** | .09 | . | . | .54 | .15 | . |
| **AN** | . | . | .54 | . | . | .05 |
| **PD** | .09 | .16 | .15 | . | . | .5 |
| **PI** | . | . | . | .05 | .5 | . |

*Note.* PB = Pain-means-harm belief, PAv = Pain Avoidance, DE = Depressive symptoms, AN = Anxious symptoms, PD = Pain Disability, PI – Pain Intensity

**Table S5**

*Partial Correlations in Extended Network*

|  | **PIt** | **PD** | **STR** | **PAf** | **FW** | **ES** | **AH** | **PIv** | **PB** | **PAc** | **PW** | **PAv** | **QoL** | **ED** | **MS** |
| --- | --- | --- | --- | --- | --- | --- | --- | --- | --- | --- | --- | --- | --- | --- | --- |
| **PIt** | . | .35 | . | . | . | . | . | . | . | . | .23 | . | . | . | . |
| **PD** | .35 | . | . | . | . | . | . | . | . | -.25 | .11 | . | -.06 | . | . |
| **STR** | . | . | . | . | . | . | -.12 | . | . | . | .08 | . | -.25 | . | .46 |
| **PAf** | . | . | . | . | .09 | .11 | . | .06 | -.06 | .07 | .1 | . | .3 | . | . |
| **FW** | . | . | . | .09 | . | . | -.12 | . | -.06 | .14 | . | . | -.23 | .14 | .21 |
| **ES** | . | . | . | .11 | . | . | . | -.18 | . | . | . | .06 | .17 | . | . |
| **AH** | . | . | -.12 | . | -.12 | . | . | -.06 | . | .11 | . | .06 | .13 | -.06 | . |
| **PIv** | . | . | . | .06 | . | -.18 | -.06 | . | . | -.08 | .18 | . | . | .1 | . |
| **PB** | . | . | . | -.06 | -.06 | . | . | . | . | . | .08 | . | . | .09 | . |
| **PAc** | . | -.25 | . | .07 | .14 | . | .11 | -.08 | . | . | -.3 | -.25 | .15 | . | . |
| **PW** | .23 | .11 | .08 | .1 | . | . | . | .18 | .08 | -.3 | . | . | -.15 | . | .16 |
| **PAv** | . | . | . | . | . | .06 | .06 | . | . | -.25 | . | . | . | . | . |
| **QoL** | . | -.06 | -.25 | .3 | -.23 | .17 | .13 | . | . | .15 | -.15 | . | . | -.2 | . |
| **ED** | . | . | . | . | .14 | . | -.06 | .1 | .09 | . | . | . | -.2 | . | . |
| **MS** | . | . | .46 | . | .21 | . | . | . | . | . | .16 | . | . | . | . |

*Note.* PIt = pain intensity, PD = pain disability, STR = stress, PAf = positive affect, FW = financial worries, ES = emotional support, AH = access to health care, PIv = pain invalidation, PB = pain-means-harm belief, PAc = pain acceptance, PW = pain-related worries, PAv = pain avoidance, QoL = quality of life, ED = experiences of discrimination, MS = mood symptoms

1. **Post-hoc network accuracy & stability**


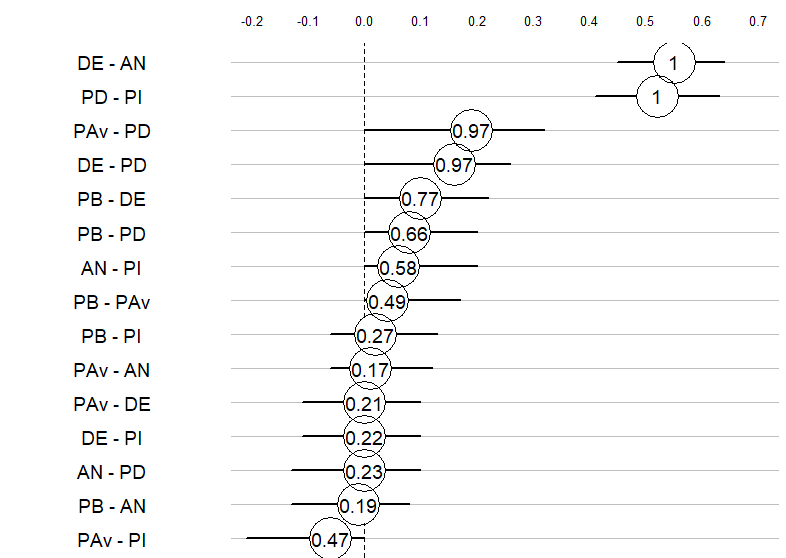


**Figure S5.** Results from the bootstrapping procedure for the replication network. Each line represents the results for one edge (estimated partial correlation). The location of the circle indicates the average estimate for that edge, while the solid line extending from the circle indicates the 95% quantiles around the estimate. The number inside the circle indicates the proportion of bootstrapped samples in which the edge was estimated to be present (e.g., 1 = 100% of the time). PB = Pain-means-harm beliefs, PAv = Pain avoidance, DE = Depressive symptoms, AN = Anxious symptoms, PD = Pain disability, PI – Pain intensity


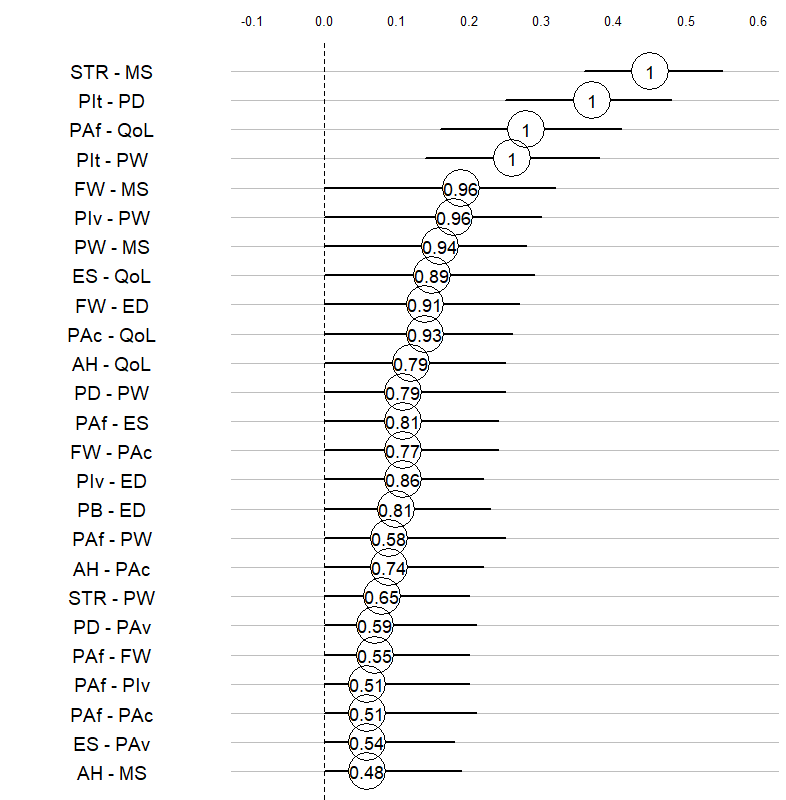


**Figure S6.** Results from the bootstrapping procedure for the 25 largest positive edges in the extended network, including the 24 edges that were estimated to be present in the main analysis. Each line represents the results for one edge (estimated partial correlation). The location of the circle indicates the average estimate for that edge, while the solid line extending from the circle indicates the 95% quantiles around the estimate. The number inside the circle indicates the proportion of bootstrapped samples in which the edge was estimated to be present (e.g., 1 = 100% of the time).


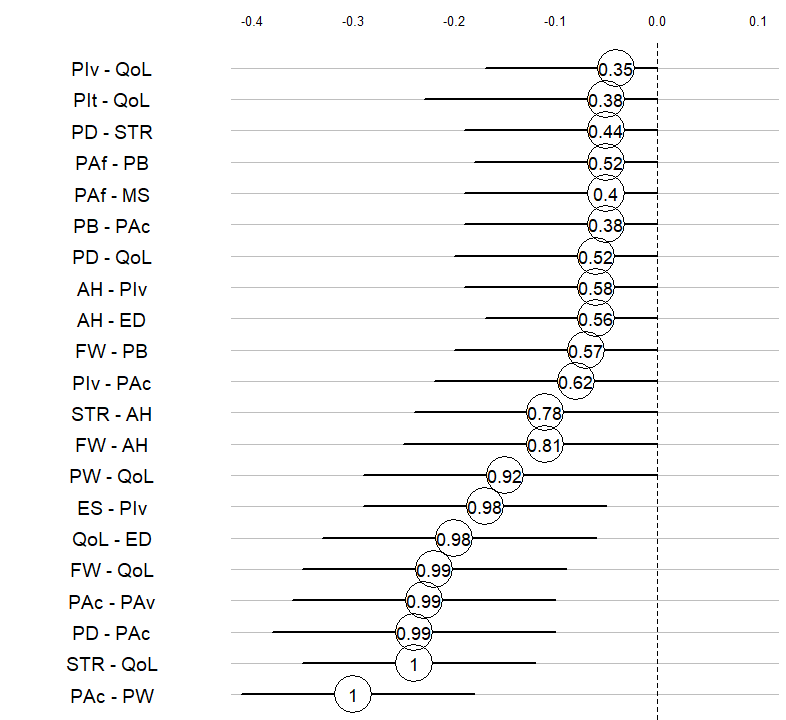


**Figure S7.** Results from the bootstrapping procedure for the 20 largest negative edges in the extended network, including the 16 edges that were estimated to be present in the main analysis. Each line represents the results for one edge (estimated partial correlation). The location of the circle indicates the average estimate for that edge, while the solid line extending from the circle indicates the 95% quantiles around the estimate. The number inside the circle indicates the proportion of bootstrapped samples in which the edge was estimated to be present (e.g., 1 = 100% of the time).

**References**

[1] Klasen BW, Hallner D, Schaub C, Willburger R, Hasenbring M. Validation and reliability of the German version of the Chronic Pain Grade questionnaire in primary care back pain patients. GMS Psycho-Social Medicine 2004;1:Doc07.

[2] Glaesmer H, Hoyer J, Klotsche J, Herzberg PY. Die deutsche version des Life-Orientation-Tests (LOT-R) zum dispositionellen Optimismus und Pessimismus. Zeitschrift Für Gesundheitspsychologie 2008;16:26–31.

[3] Klooster P ten, Weekers A, Eggelmeijer F, Van Woerkom J, Drossaert C, Taal E, et al. Optimisme en/of pessimisme: factorstructuur van de Nederlandse Life Orientation Test-Revised. Psychologie En Gezondheid 2010;38:89–100.

[4] Röcke C, Grühn D. German translation of the PANAS-X. Unpublished Manuscript, Free University Berlin 2003.

[5] Engelen, Ute, De Peuter, Steven, Victoir, An, Van Diest, Ilse, Van den Bergh O. Verdere validering van de Positive and Negative Affect Schedule (PANAS) en vergelijking van twee Nederlandstalige versies n.d.

[6] Löwe B, Wahl I, Rose M, Spitzer C, Glaesmer H, Wingenfeld K, et al. A 4-item measure of depression and anxiety: Validation and standardization of the Patient Health Questionnaire-4 (PHQ-4) in the general population. Journal of Affective Disorders 2010;122:86–95. https://doi.org/10.1016/j.jad.2009.06.019.

[7] Wingenfeld K, Spitzer C, Mensebach C, Grabe HJ, Hill A, Gast U, et al. Die deutsche version des childhood trauma questionnaire (CTQ): erste befunde zu den psychometrischen kennwerten. PPmP-Psychotherapie· Psychosomatik· Medizinische Psychologie 2010;60:442–50.

[8] Löwe B, Decker O, Müller S, Brähler E, Schellberg D, Herzog W, et al. Validation and standardization of the Generalized Anxiety Disorder Screener (GAD-7) in the general population. Medical Care 2008;46:266–74.

[9] Zenger M, Schaefert R, Van Der Feltz-Cornelis C, Brähler E, Häuser W. Validation of the Patient-Doctor-Relationship Questionnaire (PDRQ-9) in a Representative Cross-Sectional German Population Survey. PLoS ONE 2014;9:e91964. https://doi.org/10.1371/journal.pone.0091964.

[10] Van der Feltz-Cornelis CM, Van Oppen P, Van Marwijk HW, De Beurs E, Van Dyck R. A patient-doctor relationship questionnaire (PDRQ-9) in primary care: development and psychometric evaluation. General Hospital Psychiatry 2004;26:115–20.

[11] Nilges P, Köster B, Schmidt C. Schmerzakzeptanz–Konzept und Überprüfung einer deutschen Fassung des chronic pain acceptance questionnaire. Der Schmerz 2007;21:57–67.

[12] Trompetter HR, Ten Klooster PM, Köke A, Schreurs KMG. Acceptatie van pijn: problemen met de factoriële validiteit van de Nederlandse vertaling van de Chronic Pain Acceptance Questionnaire (CPAQ). PSYCHOL GEZONDH 2011;39:292–300. https://doi.org/10.1007/s12483-011-0056-y.

[13] Hotz-Boendermaker S, Tirez J, Morf R, Esteve R. Translation, reliability, and validity of the German version of the Activity Patterns Scale (APS) in musculoskeletal pain: a methodological study. BMC Musculoskelet Disord 2024;25:884. https://doi.org/10.1186/s12891-024-07986-x.

[14] Esteve R, Ramírez-Maestre C, Peters ML, Serrano-Ibáñez ER, Ruíz-Párraga GT, López-Martínez AE. Development and Initial Validation of the Activity Patterns Scale in Patients With Chronic Pain. The Journal of Pain 2016;17:451–61. https://doi.org/10.1016/j.jpain.2015.12.009.

[15] Brähler E, Mühlan H, Albani C, Schmidt S. Teststatistische prüfung und normierung der deutschen versionen des EUROHIS-QOL lebensqualität-Index und des WHO-5 wohlbefindens-index. Diagnostica 2007;53:83–96.

[16] De Vries J, Van Heck G. Nederlandse WHOQoL-Bref. Tilburg: Tilburg University 1996.
